# Pan-Atlantic analysis of the overlap of a highly migratory species, the leatherback turtle, with pelagic longline fisheries

**DOI:** 10.1098/rspb.2013.3065

**Published:** 2014-04-07

**Authors:** S. Fossette, M. J. Witt, P. Miller, M. A. Nalovic, D. Albareda, A. P. Almeida, A. C. Broderick, D. Chacón-Chaverri, M. S. Coyne, A. Domingo, S. Eckert, D. Evans, A. Fallabrino, S. Ferraroli, A. Formia, B. Giffoni, G. C. Hays, G. Hughes, L. Kelle, A. Leslie, M. López-Mendilaharsu, P. Luschi, L. Prosdocimi, S. Rodriguez-Heredia, A. Turny, S. Verhage, B. J. Godley

**Affiliations:** 1 Department of Biosciences, College of Science, Swansea University, Swansea SA2 8PP, UK; 2 Environment and Sustainability Institute, University of Exeter, Penryn Campus, Penryn TR10 9FE, UK; 3 Centre for Ecology and Conservation, University of Exeter, Penryn Campus, Penryn TR10 9FE, UK; 4 Centro de Investigación y Conservación Marina, El Pinar, Canelones 15008, Uruguay; 5 Virginia Institute of Marine Science, 1208 Greate Road, Gloucester Point, VA 23062, USA; 6 Comité Régional des Pêches et Elevages Marins de Guyane, Port de Pêche du Larivot, Matoury 97351, French Guiana; 7 Aquamarina, Del Besugo 1525, Pinamar, Buenos Aires 7167, Argentina; 8 Jardín Zoológico de la Ciudad de Buenos Aires, Republica de la India 3000, Buenos Aires 1425, Argentina; 9 Regional Program for Sea Turtles Research and Conservation of Argentina (PRICTMA) Smith 37, 1876-Bernal, Provincia de Buenos Aires, Argentina; 10 ICMBio–Reserva Biológica de Comboios, Linhares, ES 29900-970, Brazil; 11 Asociación LAST, Apdo 496-1100, Tibás, Costa Rica; 12 SEATURTLE.org, 1 Southampton Place, Durham, NC 27705, USA; 13 Dirección Nacional de Recursos Acuáticos, Constituyente 1497, Montevideo 11200, Uruguay; 14 WIDECAST, 1348 Rusticview Drive, Ballwin, MO 63011, USA; 15 Biology and Natural Resources Department, Principia College, 1 Maybeck Place, Elsah, IL 62028, USA; 16 Sea Turtle Conservancy, 4424 NW 13th St., Suite B11, Gainesville, FL 32609, USA; 17 Karumbé - Av. Rivera 3245 (Zoo Villa Dolores), Montevideo 11600, Uruguay; 18 Rue Victor Hugo, 25120 Maiche, France; 19 Wildlife Conservation Society, Global Conservation Program, 2300 Southern Boulevard, Bronx, NY 10460, USA; 20 Fundação Pró-TAMAR, Postal 2219, Rio Vermelho, Salvador, Bahia, Brazil; 21 Centre for Integrative Ecology, School of Life and Environmental Sciences, Deakin University, Warrnambool, Victoria 3280, Australia; 22 183 Amber Valley, P/Bag X30, Howick 3290, South Africa; 23 WWF Guianas, Henck Arronstraat 63 Suriname and 5 lot Katoury, Cayenne 97300, French Guiana; 24 WWF International, Avenue Mont-Blanc 27, Gland 1196, Switzerland; 25 Department of Biology, University of Pisa, Via A. Volta, 6, Pisa 56126, Italy; 26 Laboratorio Genética de la Estructura Poblacional, Departamento de Ecología, Genética y Evolución, FCEN, Universidad de Buenos Aires, Intendente Güiraldes 2160, C1428EGA Capital Federal, Buenos Aires, Argentina; 27 Fundación Mundo Marino, Avenida Décima No 157 C.C. No 6 -7105 San Clemente del Tuyú, Buenos Aires, Argentina; 28 WWF Gabon, Libreville BP 9144, Gabon

**Keywords:** incidental capture, marine protected area, international collaboration, satellite tracking, mitigation, marine vertebrate

## Abstract

Large oceanic migrants play important roles in ecosystems, yet many species are of conservation concern as a result of anthropogenic threats, of which incidental capture by fisheries is frequently identified. The last large populations of the leatherback turtle, *Dermochelys coriacea*, occur in the Atlantic Ocean, but interactions with industrial fisheries could jeopardize recent positive population trends, making bycatch mitigation a priority. Here, we perform the first pan-Atlantic analysis of spatio-temporal distribution of the leatherback turtle and ascertain overlap with longline fishing effort. Data suggest that the Atlantic probably consists of two regional management units: northern and southern (the latter including turtles breeding in South Africa). Although turtles and fisheries show highly diverse distributions, we highlight nine areas of high susceptibility to potential bycatch (four in the northern Atlantic and five in the southern/equatorial Atlantic) that are worthy of further targeted investigation and mitigation. These are reinforced by reports of leatherback bycatch at eight of these sites. International collaborative efforts are needed, especially from nations hosting regions where susceptibility to bycatch is likely to be high within their exclusive economic zone (northern Atlantic: Cape Verde, Gambia, Guinea Bissau, Mauritania, Senegal, Spain, USA and Western Sahara; southern Atlantic: Angola, Brazil, Namibia and UK) and from nations fishing in these high-susceptibility areas, including those located in international waters.

## 1. Introduction

In recent years, there has been increasing effort to sustainably manage fish populations and reverse the collapse of many target species [1]. Many non-targeted species, however, are also of conservation concern, partly owing to their incidental capture by fisheries or ‘bycatch’ [2]. Bycatch occurs globally and can particularly impact highly migratory species, whose movements can lead to an increased probability of interaction [3]. Assessing the susceptibility of such species to bycatch is challenging, as it requires an understanding of the transboundary nature of their movements, and thus requires multinational collaboration [4]. A key step forward is to map the spatio-temporal distribution of the species and the extent of interactions with fisheries (e.g. [5]). Adopting this approach generally requires large numbers of individuals to be remotely tracked, preferably from different populations and over extended periods of time, which few individual projects have achieved [6–9].

The highly migratory leatherback turtle, *Dermochelys coriacea*, is of conservation concern mainly due to the recent dramatic declines in the Pacific [10]. Today, the majority of the world's leatherback turtles occur in the Atlantic Ocean [11,12], where several rookeries have been reported to be stable or increasing [11]. Although conservation measures at sub-basin scales have been implemented [13], in both the northern and southern Atlantic bycatch in artisanal and industrial fisheries remains a major threat [3,14,15]. In the Atlantic Ocean, the scale of pelagic longline fishing effort is particularly extensive [16] and these fisheries may have a considerable impact on leatherback turtles [3,14–16]. Initial studies in the northern Atlantic have suggested that leatherbacks may be particularly at risk along dynamic oceanic fronts, where turtles feed on gelatinous plankton [17,18] and where fisheries also concentrate [19,20], although these findings are based on small sample sizes (*n* < 10 individuals). In the past decade, more than 30 satellite-tracking studies of leatherback turtles in the Atlantic Ocean have been published (see electronic supplementary material, table S1) and each of these studies has given an essential, yet partial description of habitat use.

Here, we present the first integrated analysis of the spatio-temporal distribution and habitat use of leatherback turtles between reproductive seasons at the scale of the Atlantic Ocean. This information is combined with data on the distribution of pelagic longline fishing effort obtained from the International Commission for the Conservation of Atlantic Tunas (ICCAT) across the same temporal period. This study presents a unique opportunity to identify the areas and seasons of highest susceptibility to turtle bycatch, and provides much-needed preliminary guidance on the design and implementation of potential bycatch mitigation measures at an oceanic scale.

## 2. Material and methods

### (a) Turtle-tracking dataset

Between June 1995 and February 2010, 106 platform transmitter terminals (PTTs) were deployed on leatherback turtles in the Atlantic Ocean and in the southwestern Indian Ocean (see electronic supplementary material, table S1). Our study involves an integrative synthesis of these data, which were all previously published in scientific peer-reviewed literature, except for two tracks (see electronic supplementary material, table S1). PTTs were predominantly attached to females (*n* = 101), with four on males and one on a juvenile (sex unknown). The majority of females (*n* = 93) were equipped while nesting at 13 sites fringing the Atlantic Ocean and at one site in the southwestern Indian Ocean (see electronic supplementary material, table S1), while the remaining turtles (*n* = 4 males, 8 females and 1 juvenile) were equipped at sea. Warehousing and standardization of satellite-tracking data from the research groups, which spanned 10 countries and four continents, were achieved using the Satellite Tracking and Analysis Tool (STAT) [21]. Transmissions were collected and relayed via the Argos System (https://argos-system.cls.fr). Only locations with LC (Location Classes) 3, 2, 1, A and B were used. The locations were filtered using the maximum rate of travel of 10 km h^−1^ and the maximum azimuth of 35° between successive locations [22]. The location with the greatest spatial accuracy received in each 24 h period (00.00–23.59 UTC) was then selected to minimize spatio-temporal autocorrelation in the dataset. For each turtle, when no location was received during a 24 h period, a linear interpolation was used to interpolate the route, but only for up to 5 days following the last received valid location. For turtles equipped in the nesting season, only movements recorded during the post-nesting period were used in the analysis.

#### (i) Weighting factors and normalization

##### Unequal tracking durations

No leatherback turtle has been tracked throughout a complete interbreeding migration, which is estimated to be between 1095 and 1460 days for Atlantic leatherback turtles (maximum tracking duration = 713.1 days). In order to account for (i) tracks of different durations and (ii) tracks that end near the release location, a weighting factor was applied to the tracking dataset following the method developed by Block *et al.* [6]. All tracks were normalized by weighting each location estimate by the inverse of the number of individuals that had location estimates for the same relative day of their track. We imposed a threshold relative day of tracking (85th percentile of the frequency distribution of the track lengths, i.e. 337th day) above which locations received the same weight as on the threshold day. Sixteen tracks were longer than 337 days, therefore every position after this day received a weighting of 1/16. This method, by increasing the weight of later locations and longer tracks, reduced the bias in the spatial coverage towards deployment locations.

##### Unequal sample sizes among tagging sites

The number of deployed satellite tags differed among the nesting sites and tagging effort was not proportional to the estimated number of females nesting at each site (Spearman's rank correlation, *p* = 0.086). In order to account for these unequal and unbalanced sample sizes, a second weighting factor was applied to the tracking dataset. Each rookery was assigned a weight between 0 and 1, proportional to the size of its nesting population (estimated by previous studies [23–25]) relative to the estimated total number of nesting females in the Atlantic Ocean (i.e. approx. 16 600 adult females) [23]. The Central Africa nesting assemblage was given a weight of 0.3, because the number of nesting females is estimated to be about 5000 (i.e. approx. 30% of the estimated total number of nesting females in the Atlantic Ocean) [23]. The same weight was given to the French Guiana/Suriname nesting assemblage (approx. 5000 nesting females). Weights of 0.18, 0.15, 0.04, 0.015, 0.003 and 0.003 were assigned to Trinidad and Tobago (approx. 3000 nesting females), Costa Rica/Panama (approx. 2500 nesting females), Florida (approx. 750 nesting females), Grenada (approx. 250 nesting females), South Africa (approx. 50 nesting females) and Brazil (approx. 50 nesting females) nesting assemblages, respectively.

Thirteen turtles were equipped with satellite tags on their foraging grounds and therefore could not be directly attributed to any particular nesting assemblage. We therefore attributed each of these tracks to the most likely nesting assemblage, based on the best scientific information available. Seven turtles were captured in the southwestern Atlantic Ocean. Considering that the main nesting population in the southern Atlantic is the Central African population (5000 females versus 50 females in Brazil), we attributed all seven tracks to the former and gave them a weight of 0.3. Two turtles were captured at sea off Ireland. The origin of leatherback turtles foraging in the northeast Atlantic has not yet been investigated, therefore we arbitrarily, but conservatively, attributed both tracks to the French Guiana nesting assemblage and weighted them accordingly. Four turtles were captured at sea off the Atlantic coast of Canada. A recent study investigating the origin of leatherback turtles foraging in Canadian waters [26] allowed us to attribute two tracks to the French Guiana assemblage, one track to the Trinidad and Tobago assemblage and one track to the Costa Rica/Panama nesting assemblage, and gave weights of 0.3, 0.18 and 0.15, respectively.

The weighting process ensured that tracks from larger nesting assemblages contributed a higher weight in subsequent density mapping than those from smaller nesting assemblages, even though tagging effort was disproportionate among the nesting sites.

#### (ii) Mapping of turtle distribution

A density map was constructed for each nesting assemblage from filtered, tracking-duration-weighted location data. The population size-weighting process was applied to each nesting-assemblage-specific spatial density map. The maps from each nesting assemblage were then summed to estimate areas of high use. Three occupancy classes were defined, and therefore three types of areas: low- (less than 25th percentile), medium- (more than or equal to 25th and less than 75th percentile) and high-use areas (more than or equal to 75th percentile). Following the same method, maps were also constructed for each quarter (i.e. January–March, April–June, July–September and October–December).

### (b) Fisheries dataset

All spatio-temporally relevant pelagic longline fishing effort data from the T2CE (Task II Catch and Effort) database from the ICCAT were utilized (1995–2009; northern and southern Atlantic). Fisheries data were prepared at monthly intervals to a spatial resolution of 5 × 5°. Only the records of fishing-effort reporting the number of hooks deployed were considered, as effort reported using other units was estimated to account for less than 2% of the total effort. Three fishing-effort classes were defined: low (less than 25th percentile, i.e. less than 7375 hooks km^−2^), medium (more than or equal to 25th and less than 75th percentile, i.e. 7375 ≤ medium < 58 748 hooks km^−2^) and high (more than or equal to 75th percentile, i.e. 58 848 ≤ high < 415 757 hooks km^−2^). Three classes representing the consistency in fishing effort were also defined: constant (less than 25th percentile), moderate (more than or equal to 25th and less than 75th percentile) and variable effort (more than or equal to 75th percentile). Three classes representing the fishery pressure were subsequently defined based on a combination of the three fishing-effort classes and the three classes representing consistency in fishing effort. This pressure index had three levels: low, medium and high pressure. For a given level of fishing effort (low, medium or high), we assumed that constant (i.e. sustained) fishing effort has more impact on a species or an ecosystem than variable (i.e. irregular) fishing effort (e.g. [27]). Therefore areas with the following combinations of fishing effort and fishing consistency classes received a low-pressure index: low/variable, low/moderate or medium/variable fishing effort. Areas with low/constant, medium/moderate and high/variable fishing effort and fishing consistency classes had a medium-pressure index; and areas with medium/constant, high/moderate and high/constant fishing effort and fishing consistency classes were classified as high-pressure index. The pressure index was also collated into quarters (i.e. January–March, April–June, July–September and October–December).

### (c) Leatherback spatio-temporal susceptibility to longline fisheries bycatch

In order to assess spatial and temporal variation of leatherback susceptibility to longline fisheries bycatch, we first selected areas having a high fishing-pressure index, both annually and for each quarter separately. We then categorized these areas based on the coincident annual and seasonal estimates of leatherback turtle density. Areas of high fishing pressure coincident with high turtle density were classified as ‘high’ susceptibility, areas of high fishing pressure and medium turtle density were classified as ‘medium’ susceptibility, and areas of high fishing pressure and low turtle density were classified as ‘low’ susceptibility. Maps overlaying areas of leatherback habitat use with (i) areas having a medium fishing-pressure index (see electronic supplementary material, figure S6A) or (ii) areas having a low fishing-pressure index (see electronic supplementary material, figure S6B) were also generated for comparison.

Data were analysed and mapped using Matlab (The MathWorks, MA), the R software package [28] and ArcGIS v. 10.1 and 10.5 (Environmental Systems Research Institute, Redlands, CA).

## 3. Results

**Figure 1.**
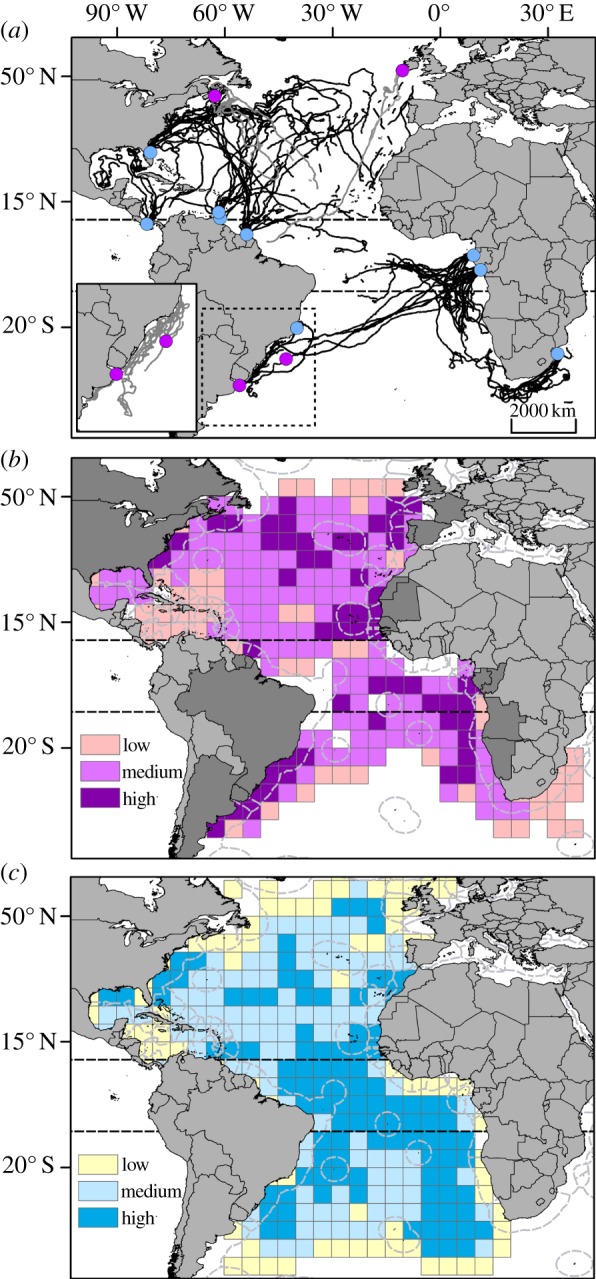
Movements and density distribution of satellite-tracked leatherbacks and pelagic longline fishing-pressure index in the Atlantic Ocean over 15 years. (*a*) Movements of satellite-tracked leatherbacks during their migration in the Atlantic Ocean, between 1995 and 2010. Black lines: movements of females tagged on the nesting beach (*n* = 93). Grey lines: movements of individuals tagged near presumed foraging grounds (*n* = 13; four males, one juvenile and eight females). Blue dots: deployment from a nesting site. Purple dots: deployment at sea (see the electronic supplementary material, table S1). Inset: movements of six individuals tagged on their foraging grounds in the southwestern Atlantic. (*b*) Density of leatherback daily locations (locations were time-weighted and population-size-normalized). Three density classes were defined: low, medium and high use. White pixels represent areas from which tracking data were not received. High-use areas occurred both in international waters and within the EEZs of 20 countries (in dark grey) fringing the northern Atlantic (Canada, Cape Verde, Gambia, Guinea Bissau, France/French Guiana, Mauritania, Portugal/Azores, Senegal, Spain/Canaries, Suriname, United States of America, Western Sahara) or the southern Atlantic (Angola, Argentina, Brazil, Congo, Gabon, Namibia, United Kingdom/Ascension Island and Uruguay). Dashed grey lines represent the limits of national EEZs. (*c*) Fishing-pressure index for the period 1995–2009 in the Atlantic Ocean. This index resulted from the combination of the three fishing-effort classes (see electronic supplementary material, figure S2B) and the three consistency-in-fishing-effort classes (see electronic supplementary material, figure S3B). This index had three levels of increasing intensity (low, medium and high; see Material and methods for more detail). Broken lines represent latitudes 10° N and 10° S.

Between 1995 and 2010, 106 turtles were satellite-tracked from sites throughout the Atlantic Ocean and the southwestern Indian Ocean (figure 1*a*) for a duration varying between six and 713 days (see electronic supplementary material, table S1). Individuals rarely moved between the Northern and Southern hemispheres (figure 1*a*), allowing us to define two regional management units [29] with some confidence: northern and southern Atlantic (the latter including turtles from South Africa).

Maps of daily turtle locations revealed that Atlantic leatherbacks use both offshore international waters and coastal national waters, either seasonally or year-round, leading to a complex pattern of spatio-temporal habitat use (figure 1*b*; electronic supplementary material, figure S1). Turtles used the exclusive economic zones (EEZs) of 46 out of the 97 (47.0%) countries bordering the Atlantic Ocean (figure 1*b*). In the northern Atlantic, 53.0% of all daily locations were located in international waters and 47.0% in EEZs (*n* = 6863 locations for 65 turtles), compared with 54.5% and 45.5%, respectively, in the southern Atlantic (*n* = 5664 locations for 50 turtles).

In the northern Atlantic, despite all breeding being in the west, high-use areas mainly occurred in the central (25–50° N, 50–30° W) and eastern regions, and in particular in the waters offshore western Europe, around Cape Verde (year-round) and around the Azores (October–March). High-use areas also occurred along the east coast of the USA (April–June and October–December) and off Canada (July–December; figure 1*b*; electronic supplementary material, figure S1; see also [18,30,31]). A relatively broad migratory corridor was visible when turtles departed their nesting sites in French Guiana/Suriname and their movements overlapped with turtles from Grenada and Trinidad (July–September; figure 1*a*,*b*; electronic supplementary material, figure S1).

In the southern Atlantic, leatherbacks leaving their nesting sites in Gabon displayed a narrower range in distribution and appeared to use a migratory corridor towards the coast of South America (January–March; see also [12]). Along the coast of South America, movements of turtles tracked from the southwestern Atlantic feeding grounds and Brazilian rookeries overlapped with those from Gabon, resulting in a year-round high-use area occurring from 20°S to 45° S (figure 1*a*,*b*; see also [12,32,33]). Two other high-use areas occurred: one in the equatorial central Atlantic (April–September) and one off the west coast of southern Africa (5–30° S; April–June and October–December). In this latter area, turtles tracked from Gabon and South Africa (southwestern Indian Ocean) overlapped (figure 1*a*,*b*; electronic supplementary material, figure S1).

More than four billion hooks were set throughout the Atlantic by pelagic longline fisheries between 1995 and 2010, equivalent to roughly 730 000 hooks d^−1^. By combining data on the magnitude (see electronic supplementary material, figure S2) and inter-annual variation (see electronic supplementary material, figure S3) of fishing effort, an index of fishing pressure was calculated (figure 1*c*; see Material and methods). Fishing pressure was high (63% of the fished area) year-round in the equatorial central Atlantic (i.e. between 10° N and 10° S). In the northern Atlantic (more than 10° N), fishing pressure was high in 28% of the fished area compared with 43% in the southern Atlantic (more than 10° S) (figure 1*c*), with important seasonal variations in both cases (see electronic supplementary material, figure S4).

**Figure 2.**
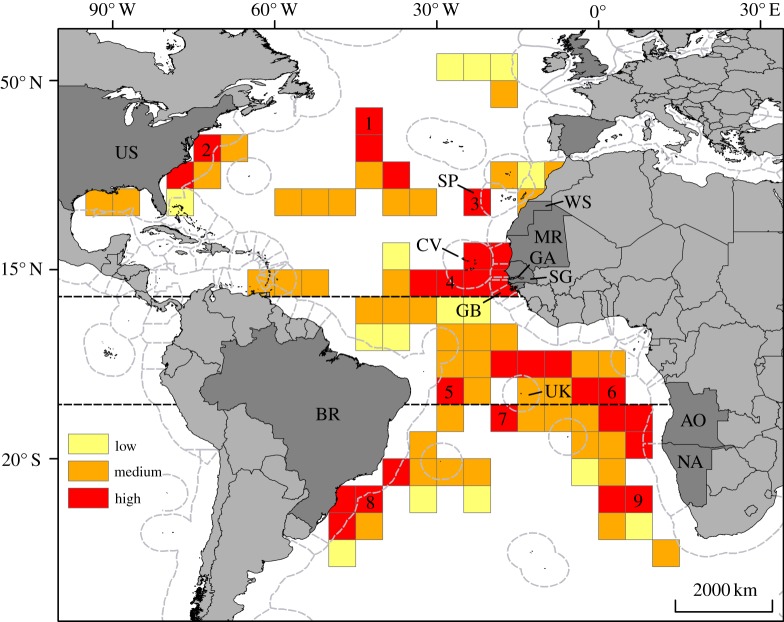
Long-term susceptibility of leatherback turtle to bycatch in longline fisheries. This map shows where high-fishing-pressure areas overlapped with leatherback habitat use, between 1995 and 2010, in the Atlantic Ocean. Three classes were defined: low (high fishing pressure/low turtle use), medium (high fishing pressure/medium turtle use) and high susceptibility (high fishing pressure/high turtle use). Nine main high-susceptibility areas were identified (nos 1–9 on the map). These areas occurred both in international waters and in the EEZs of 12 countries (in dark grey) fringing the Atlantic, comprising eight in the northern Atlantic—Cape Verde (‘CV’, no. 4), Gambia (‘GA’, no. 4), Guinea Bissau (‘GB’, no. 4), Mauritania (‘MR’, no. 4), Senegal (‘SG’, no. 4), Canaries (Spain; ‘SP’, no. 3), United States of America (‘US’, no. 2), Western Sahara (‘WS’, no. 4)—and four in the southern Atlantic—Angola (‘AO’, no. 6), Brazil (‘BR’, nos. 5 and 8), Namibia (‘NA’, no. 6), Ascension Island (United Kingdom; ‘UK’, nos. 6 and 7). Dashed grey lines represent the limits of national EEZs. Broken lines represent latitudes 10° N and 10° S.

The spatio-temporal susceptibility of leatherbacks to potential bycatch in longline fisheries was assessed (see Material and methods) by overlaying areas of leatherback habitat use (figure 1*b*) with high-fishing-pressure areas (figure 1*c*). In the northern Atlantic, a total of four seasonal high-susceptibility areas were identified: one in the central northern Atlantic in international waters, one along the east coast of the United States of America, and one each in the Canary and Cape Verdean basins (figure 2; electronic supplementary material, figure S5). These areas partly occurred in the EEZs of eight countries (Cape Verde, Gambia, Guinea Bissau, Mauritania, Senegal, Spain/Canaries, United States of America and Western Sahara; figure 2; electronic supplementary material, figure S5). In the southern Atlantic, five high-susceptibility areas were identified (equatorial: *n* = 2; temperate: *n* = 3). A high-susceptibility area located along the southern coast of Brazil persisted year-round, while others located in the equatorial central Atlantic and the Guinea, Angola and Cape basins were seasonal (figure 2; electronic supplementary material, figure S5). One area was located in international waters while the others partially or entirely occurred in the EEZs of four countries (Angola, Brazil, Namibia and United Kingdom/Ascension Island; figure 2; electronic supplementary material, figure S5). In eight of these nine high-susceptibility areas, bycatch of leatherbacks by pelagic longline fisheries has been reported [3,14–16,34,35], the only exception being around Cape Verde (i.e. area 4, figure 2).

## 4. Discussion

Our results highlight the plasticity and diversity in the spatio-temporal distribution patterns of Atlantic leatherback turtles. While several high-use areas identified in this study have been previously described [12,33,36] or tentatively suggested [18,30,31], including the migratory corridor offshore Gabon (from January to March [12]), the migratory corridor detected offshore French Guiana (from July to September) is highlighted for the first time. Caution is needed, however, as no individual track spanned the entire interbreeding period and the number of tracks in any specific area remained limited. There could therefore be high-use areas as yet undescribed.

This study highlights the transboundary nature of the leatherback distribution and movements, and the multinational effort that will be necessary to design and monitor protection measures for this species [4]. Our results specially warrant consideration by Regional Fisheries Management Organizations and the need for subsequent actions to limit the potential for bycatch, by prioritizing specific areas and times where bycatch of leatherbacks needs to be assessed and mitigated. We suggest that the high-susceptibility areas identified in this study be considered as candidates for this approach. These areas were located in both international waters and at least 12 national EEZs, and were of varying size, suggesting that different challenges might be associated with their management. For instance, the areas located in the Guinea and Angola basins (see also [15]) were extremely broad in extent, while the area located around the Canaries (January–March) or the area off the coast of southwest Africa (April–September) was much narrower. In broader areas, gear modifications and alternative fishing practices [37] may be more effective in reducing bycatch than marine protected areas or temporary spatial closures. Nonetheless, the latter have proved to be more successful for spatially smaller seasonal areas [38] and the Canary Islands, for instance might be suitable candidates for this strategy [15]. Organizations such as ICCAT might help to coordinate multinational bycatch mitigation strategies, in particular in high-susceptibility areas located in international waters [13].

A similar analysis to ours has been undertaken in the Pacific Ocean [39]. While a direct comparison of the extent and number of high-susceptibility areas in both oceans is difficult owing to differing methodologies, it appears that high-susceptible areas in the Atlantic occur to a greater extent within national EEZs. High-susceptibility areas located in national EEZs may be better candidates for management, as mitigation strategies would need to involve only a single government and a potentially limited number of fleets [13]. However, integrated approaches to ecosystem management and bycatch mitigation would need to be developed to balance ecological and economic objectives over the long term (e.g. [40]). Some nations have already implemented management actions in their EEZs to reduce turtle bycatch in pelagic longlines. Yet few or no regulations are in place in many parts of the Atlantic Ocean, and regulations are particularly lacking in many parts of the southern Atlantic where, according to our study, the majority of high-susceptibility areas might occur.

In the Atlantic Ocean, leatherback turtles travel and forage at varying depths depending on local oceanographic conditions and vertical prey distribution [18,41,42]. They spend the majority of their time, however, in the upper 200 m [43]. This flexible diving behaviour suggests that leatherback turtles are likely to encounter pelagic longlines throughout the Atlantic, whether they are predominantly engaged in foraging or post-nesting migration. Our analysis therefore did not take the behavioural states of the tracked turtles (i.e. foraging versus travelling) into account. In addition, leatherbacks incidentally captured by longlines often get entangled in the lines themselves [44,45], reinforcing that interactions can potentially happen anywhere in the upper water column (where the longlines and associated components are found), and not only at the depths (where hooks remain during soak time).

Globally, wider availability of bycatch rates, in combination with increased transparency and stricter rules for the reporting of bycatch and fishing effort by all fisheries, would greatly help in the assessment of bycatch risks and the design of effective mitigation for species of conservation concern. Besides pelagic longline fisheries, other fisheries employing different gear, such as gillnet and trawl fisheries, can also have high leatherback turtle bycatch rates [3]. Fishing-effort datasets at the scale of the Atlantic and over the 15-year period considered in this study are, however, only available for pelagic longline fisheries. The primary goal of our analysis was to identify the areas and seasons of highest susceptibility to turtle bycatch. We therefore chose to focus our analysis on the pelagic longline fishery owing to its ubiquity throughout the Atlantic, its known potential to affect leatherback populations [16] and its uniqueness regarding data availability.

While our study was successful in describing specific areas and seasons where bycatch susceptibility is high, a finer temporal and spatial resolution of fishing-effort data could undoubtedly enhance our findings. It should also be noted that the existence of illegal, unreported and unregulated (IUU) fishing is another important factor, which has not yet been reliably assessed (e.g. [46]). Additionally, the impact of coastal fisheries, even though difficult to quantitatively assess, must not be overlooked, particularly in the Atlantic, where leatherbacks use coastal and near-coastal areas (e.g. [47]).

This study offers clear pathways forward to improve the conservation status of this iconic species. The collaboration of many data providers, facilitated by the use of the online data warehouse STAT [21], has allowed the assembly of this tracking dataset for Atlantic leatherbacks to unprecedented magnitude. Additional tagging efforts, targeting specific sex and age classes, and filling geographic gaps of known foraging and breeding hotspots (e.g. [48,49]), remain important to further improve the understanding of leatherback habitat use and bycatch susceptibility. However, significant efforts are urgently needed to bridge the gap between scientists and the fishing industry to ensure that these and future findings are rapidly progressed into policy.

## Supplementary Material

Fossette_Witt et al_Figures S1 to S6_Table S1

## Data accessibility

Tracking data are stored on www.seaturtle.org. Requests for materials should be addressed to sabrina.fossette@googlemail.com or m.j.witt@exeter.ac.uk. Data are also available from the Dryad Digital Repository: http://doi.org/10.5061/dryad.h21d3.
