# Supplementary material for: Pan-Atlantic analysis of the overlap of a highly migratory species, the leatherback turtle, with pelagic longline fisheries
Source: Proc Biol Sci. 2014 Apr 7;281(1780):20133065. doi: 10.1098/rspb.2013.3065 (PMC4027393; doi:10.1098/rspb.2013.3065)
Supplement: Fossette_Witt et al_Figures S1 to S6_Table S1 [file rspb20133065supp1.pdf]

SUPPLEMENTARY INFORMATION

Pan-Atlantic analysis of the overlap of a highly migratory species, the leatherback turtle, with pelagic longline fisheries

Fossette S.*, Witt M.J.*, Miller P., Nalovic M.A., Albareda D., Almeida A.P., Broderick A.C., Chacón-Chaverri D., Coyne M.S., Domingo A., Eckert S., Evans D., Fallabrino A., Ferraroli S., Formia A., Giffoni B., Hays G.C., Hughes G., Kelle L., Leslie A., López-Mendilaharsu M., Luschi P., Prosdocimi L., Rodriguez-Heredia S., Turny A., Verhage S. and Godley B.J.

* Corresponding author(s): sabrina.fossette@googlemail.com ; m.j.witt@exeter.ac.uk

1- Supplementary Figures

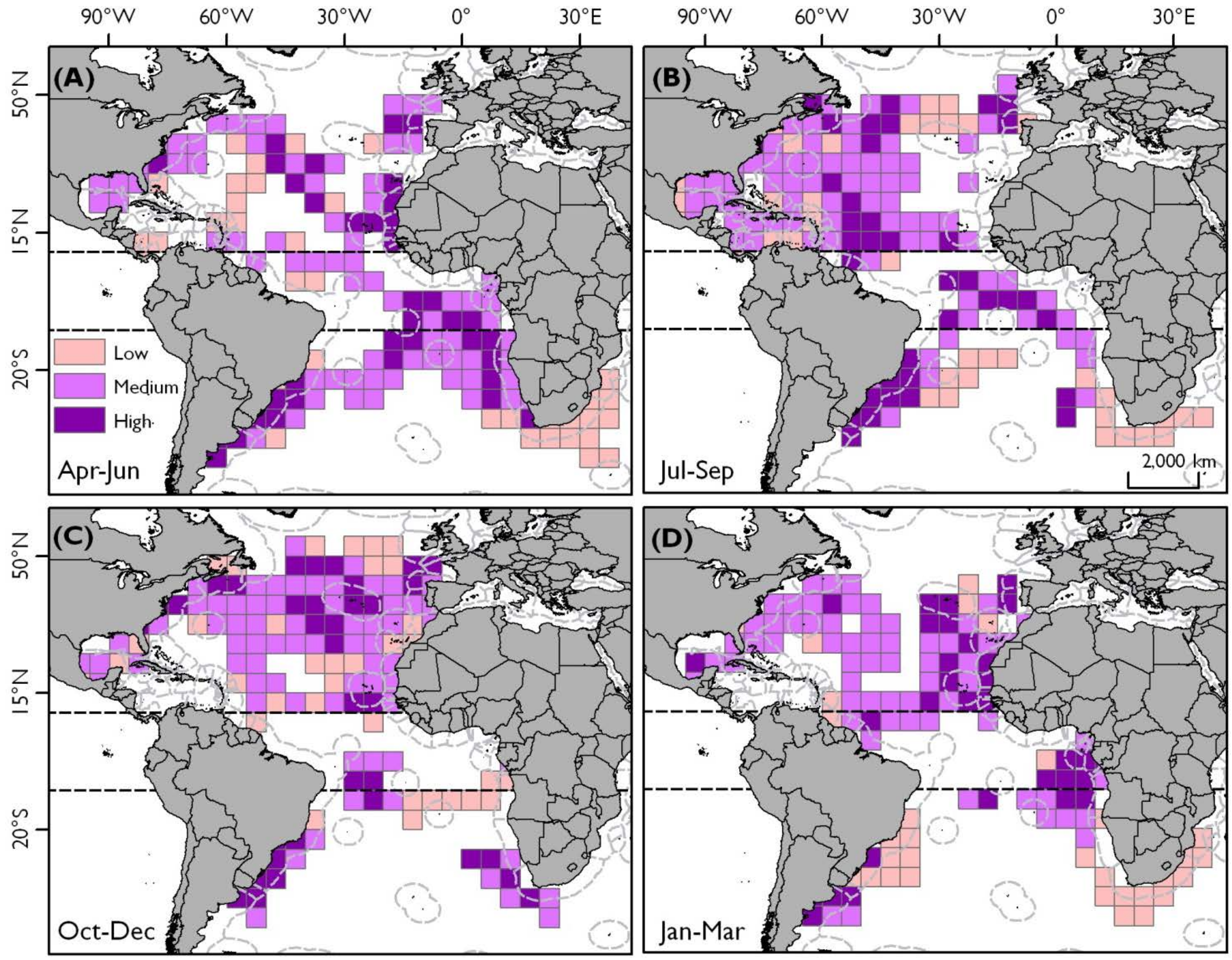

Figure S1: Density of leatherback turtle daily locations stratified by annual quarter. Locations were time-weighted and population-size normalized, then summed within 5×5° grid cells. Three density classes were defined: low, medium, and high use. White pixels represent areas for which no tracking data were received during the corresponding quarter. Dashed grey lines represent the limits of national Exclusive Economic Zones (EEZs). Broken lines: latitudes 10°N and 10°S.

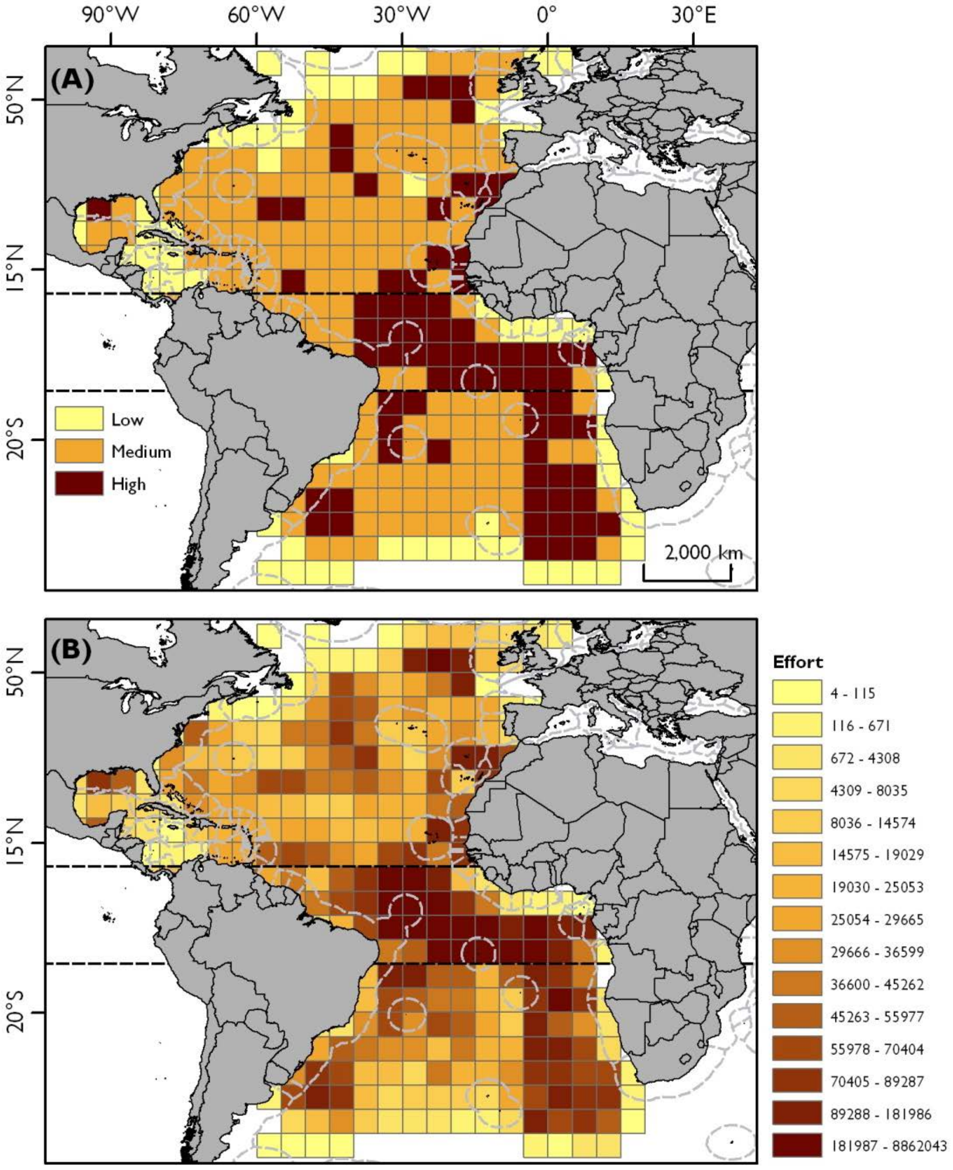

Figure S2: Pelagic longline fishing effort aggregated for all nations, from 1995 to 2009, in the Atlantic Ocean. (A) Three fishing effort classes were defined: low < 7 375 hooks per km^2, 7 375 ≤ medium < 58 748 hooks per km^2, high ≥ 58 848 hooks per km^2. (B) Effort was defined as actual number of longline hooks set per 5×5° grid cell.

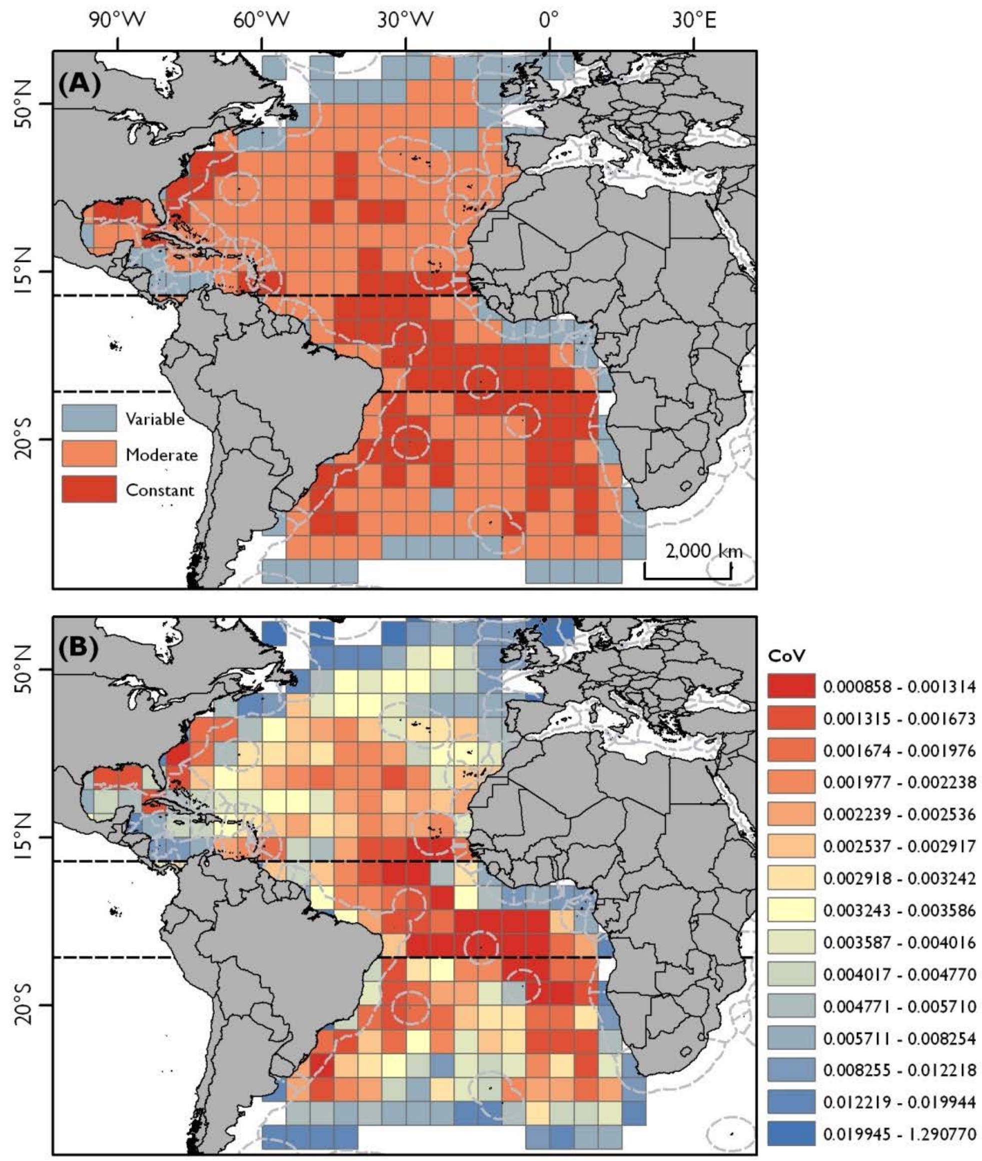

Figure S3: Inter-annual coefficient of variation (CoV) of pelagic longline fishing effort, from 1995 to 2009, in the Atlantic Ocean. Values were calculated for each 5×5° grid cell. (A) Three consistency-in-fishing-effort classes were defined: variable, moderate, and constant. (B) CoV was defined as actual values. Broken lines: latitudes 10°N and 10°S. Dashed grey lines represent the limits of national EEZs.

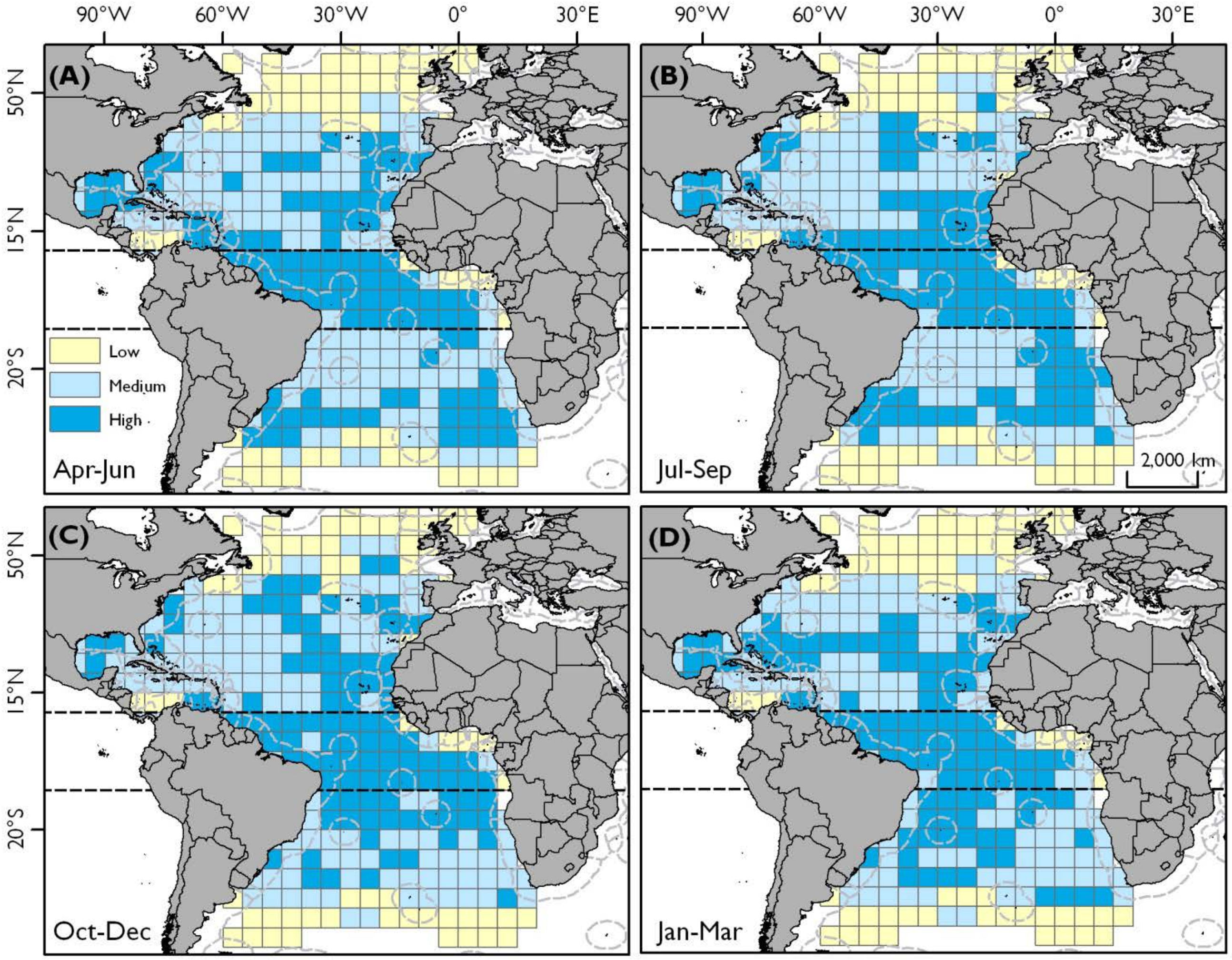

Figure S4: Fishing pressure index for the period 1995-2009 in the Atlantic Ocean, stratified by annual quarter. This index resulted from the combination of the three fishing-effort classes and the three consistency-in-fishing-effort classes, and was calculated for each 5 × 5° grid cell. This index had three levels of increasing intensity: low, medium, and high. Dashed grey lines represent the limits of national EEZs. Broken lines: latitudes 10°N and 10°S.

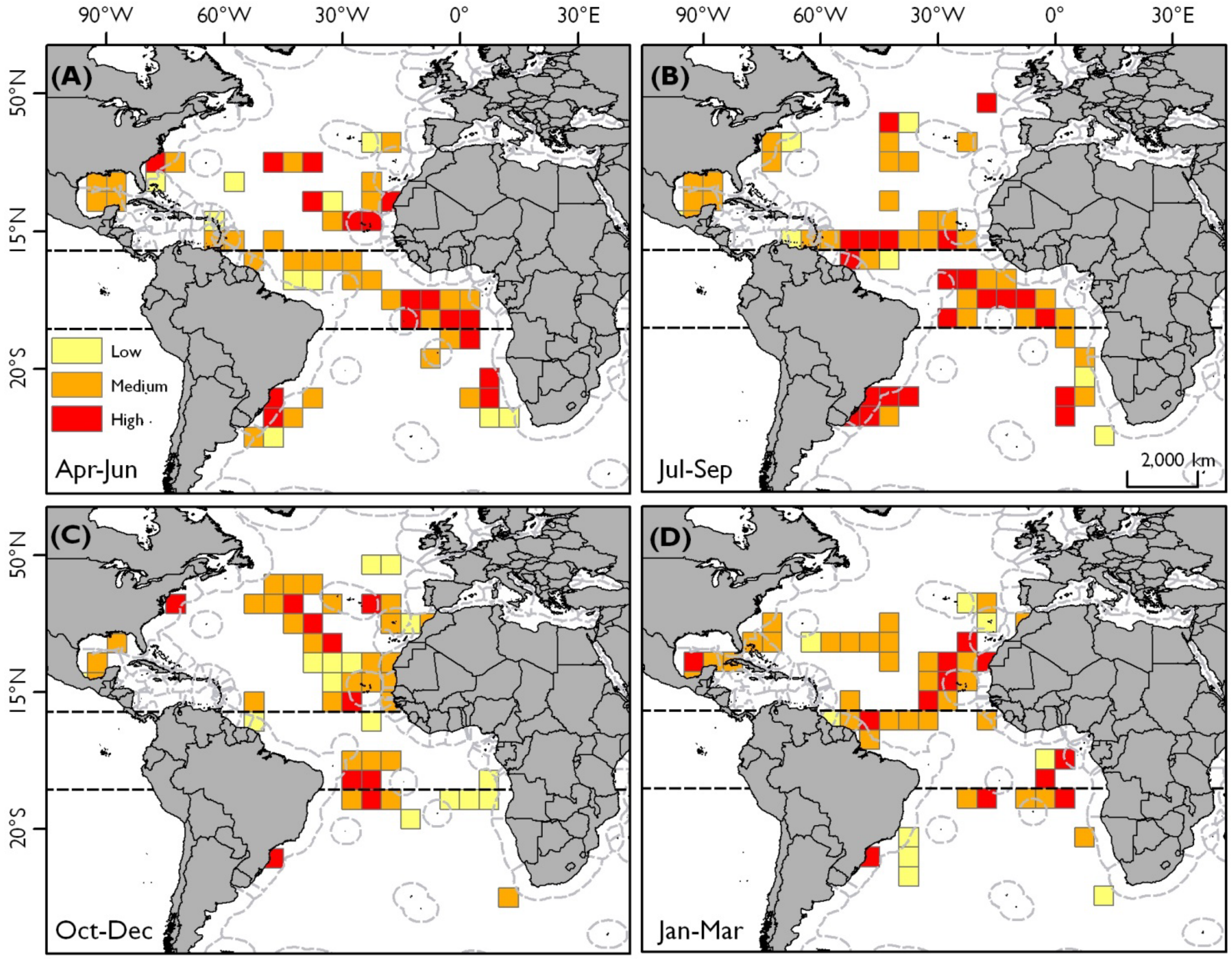

Figure S5: Spatio-temporal susceptibility of leatherback turtles to longline fisheries bycatch. These maps show for each annual quarter where high fishing-pressure areas overlapped with leatherback habitat use, between 1995 and 2010, in the Atlantic Ocean. Three susceptibility classes were defined: low susceptibility (high fishing pressure/low leatherback use), medium susceptibility (high fishing pressure/ medium turtle use), and high susceptibility (high fishing pressure/high turtle use). Dashed grey lines represent the limits of national EEZs. Broken lines: latitudes 10°N and 10°S.

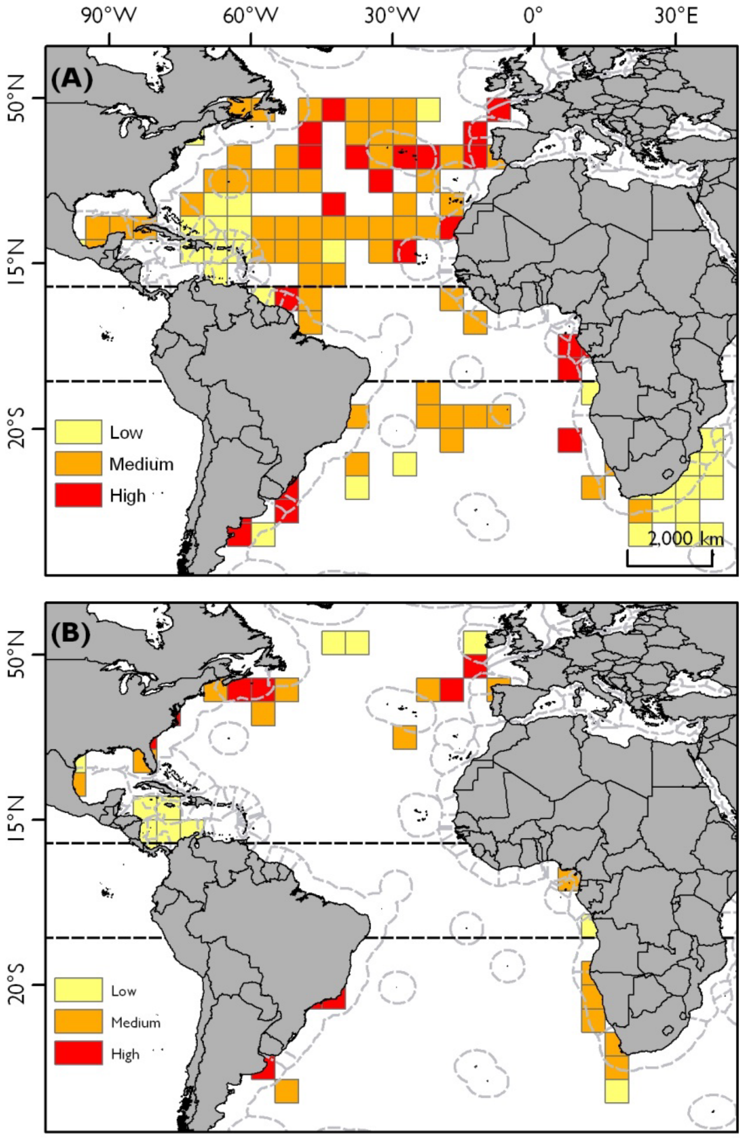

Figure S6: Long-term susceptibility of leatherback turtle to bycatch in longline fisheries. (A) This map shows where medium fishing-pressure areas overlapped with leatherback habitat use, between 1995 and 2010, in the Atlantic Ocean. Three classes were defined: low susceptibility (medium fishing pressure/low turtle use), medium susceptibility (medium fishing pressure/medium turtle use), and high susceptibility (medium fishing pressure/high turtle use). (B) This map shows where low fishing-pressure areas overlapped with leatherback habitat use, between 1995 and 2010, in the Atlantic Ocean. Three classes were defined: low susceptibility

(low fishing pressure/low turtle use), medium susceptibility (low fishing pressure/medium turtle use), and high susceptibility (low fishing pressure/high turtle use). Dashed grey lines represent the limits of national EEZs. Broken lines represent latitudes 10°N and 10°S.

2- Supplementary Table S1

Summary of tracking data for leatherback turtles in the Atlantic Ocean. All turtles stopped transmitting before 2010 which the exception of three individuals: two turtles tracked from Gabon, whose PTTs ceased transmitting in April and June 2010 and a single turtle from Argentina (instrumented in February 2010) that transmitted until July 2011.

Country of deployment	Deployment years	Turtles (n)	Mean duration (Min/Max) in days	References
Argentina	2010	1	448.4	This study
Brazil	2005-2006	4	232.8 (28.1/410.1)	31
Canada	2005-2006	4	134.6 (71.9/279.1)	18, 45, 46
Costa Rica	2004;2009	3	16.5 (9.7/20.8)	32
French Guiana/Suriname	1999- 2003; 2005	16	194.2 (22.0/713.1)	19, 18, 45, 47-49
Gabon	2003-2004; 2006; 2008-2010	31	142.7 (5.7/502.9)	12, 45
Grenada	2002-2003	9	305.0 (175.3/432.0)	20, 18, 46, 40, 50-52
Ireland	2005-2006	2	270.5 (231.1/309.9)	18, 46, 51
Panama	2005-2006	6	293.8 (30.2/553.2)	30, 45
South Africa	1996; 1999-2003	9	135.0 (48.8/246.1)	39, 53-58
Trinidad and Tobago	1995; 2002-2003	6	183.2 (18.1/369.0)	29
United States of America	2000-2002	10	185.5 (27.8/355.9)	59
Uruguay and International waters	2005-2006; 2008	5	295.0 (37.9/628.5)	32, 45, this study

3- Supplementary References

45. Fossette, S., Girard, C., López-Mendilaharsu, M., Miller, P., Domingo, A., Evans, D., Kelle, L., Plot, V., Prosdocimi, L., Verhage, S., et al. 2010 Atlantic leatherback migratory paths and temporary residence areas. *PLoS ONE* **5**, e13908.

46. Bailey, H., Fossette, S., Bograd, S.J., Shillinger, G.L., Swithenbank, A.M., Georges, J.-Y., Gaspar, P., Strömberg, K.P., Paladino, F.V. & Spotila J.R. 2012 Movement patterns for a critically endangered species, the leatherback turtle (*Dermochelys coriacea*), linked to foraging success and population status. *PloS ONE* **7**, e36401.

47. Fossette, S., Corbel, H., Gaspar, P., Le Maho, Y. & Georges, J.Y. 2008 An alternative technique for the long-term satellite tracking of leatherback turtles. *Endang. Species Res.* **4**, 33-41.

48. Gaspar, P., Georges, J.Y., Fossette, S., Lenoble, A., Ferraroli, S. & Le Maho, Y. 2006 Marine animal behaviour: neglecting ocean currents can lead us up the wrong track. *Proc. R. Soc. Lond. B.* **273**, 2697-2702.

49. Caut, S., Fossette, S., Guirlet, E., Angulo, E., Das, K., Girondot, M. & Georges, J.Y. 2009 Correction: Isotope Analysis Reveals Foraging Area Dichotomy for Atlantic Leatherback Turtles. *PloS ONE* **3**, e1845.

50. Hays, G.C., Houghton, J.D.R., Isaacs, C., King, R.S., Lloyd, C. & Lovell, P. 2004 First records of oceanic dive profiles for leatherback turtles, *Dermochelys coriacea*, indicate behavioural plasticity associated with long-distance migration. *Anim. Behav.* **67**, 733-743.

51. Doyle, T.K., Houghton, J.D.R., O'Suilleabhain, P.F., Hobson, V.J., Marnell, F., Davenport, J. & Hays, G.C. 2008 Leatherback turtles satellite-tagged in European waters. *Endang. Species Res.* **4**, 23-31.

52. McMahon, C.R. & Hays, G.C. 2006 Thermal niche, large-scale movements and implications of climate change for a critically endangered marine vertebrate. *Global Change Biol.* **12**, 1330-1338.

53. Luschi, P., Hays, G.C. & Papi, F. 2003 A review of long-distance movements by marine turtles, and the possible role of ocean currents. *Oikos* **103**, 293-302.

54. Luschi, P., Lutjeharms, J.R.E., Lambardi, P., Mencacci, R., Hughes, G.R. & Hays, G.C. 2006 A review of migratory behaviour of sea turtles off southeastern Africa. *S. Afr. J. Sci.* **102**, 51-58.

55. Luschi, P., Sale, A., Mencacci, R., Hughes, G.R., Lutjeharms, J.R.E. & Papi, F. 2003 Current transport of leatherback sea turtles (*Dermochelys coriacea*) in the ocean. *Proc. R. Soc. Lond. B.* **270**, S129-S132.

56. Lambardi, P., Lutjeharms, J.R.E., Mencacci, R., Hays, G.C. & Luschi, P. 2008 Influence of ocean currents on long-distance movement of leatherback sea turtles in the Southwest Indian Ocean. *Mar. Ecol. Prog. Ser.* **353**, 289-301.

57. Sale, A. & Luschi, P. 2009 Navigational challenges in the oceanic migrations of leatherback sea turtles. *Proc. R. Soc. Lond. B.* **276**, 3737-3745.

58. Galli, S., Gaspar, P., Fossette, S., Calmettes, B., Hays, G.C., Lutjeharms, J.R.E. & Luschi, P. 2012 Orientation of migrating leatherback turtles in relation to ocean currents. *Anim. Behav.* **84**, 1491-1500.

59. Eckert, S.A., Bagley, D., Kubis, S., Ehrhart, L., Johnson, C., Stewart, K. & DeFreese, D. 2006 Internesting and postnesting movements and foraging habitats of leatherback sea turtles (*Dermochelys coriacea*) nesting in Florida. *Chelonian Conserv. Biol.* **5**, 239-248.
